# Comment on: Effects of time of initiation of antiretroviral therapy in the treatment of patients with HIV/TB co-infection, by Chelkeba L. et al

**DOI:** 10.1016/j.amsu.2020.06.039

**Published:** 2020-07-11

**Authors:** François-Xavier Blanc, Didier Laureillard, Anne E. Goldfeld

**Affiliations:** a Department of Respiratory Medicine, L'Institut du Thorax, Nantes University Hospital, & Medical School, University of Nantes, Nantes, France; b Department of Infectious and Tropical Diseases, Nîmes University Hospital, Nîmes, France; c Pathogenesis and Control of Chronic Infections, INSERM, EFS, Montpellier University, Montpellier, France; d Program in Cellular and Molecular Medicine, Boston Children's Hospital, Boston, MA, USA

Dear Editor,

The recent review and meta-analysis by Chelkeba L. et al. erroneously claims in its abstract that ‘Early antiretroviral therapy (ART) was associated with overall mortality compared with late ART initiation’ [1]. This conclusion is not concordant with the meta-analysis in the manuscript, which showed that early ART initiation was associated with a lower overall mortality rate when compared to late ART initiation (risk ratio = 0.81; 95% confidence interval: 0.66–0.99, p = 0.04). This error could lead to delaying ART for tuberculosis (TB)-HIV co-infected patients, leading to avoidable increased mortality.

This meta-analysis aimed to analyze tuberculosis-associated immune reconstitution inflammatory syndrome (TB-associated IRIS) and mortality with regard to the timing of ART initiation in HIV-infected adult and adolescent non-pregnant patients from 8 randomized controlled trials published between 2009 and 2015. It showed that early ART initiation was associated with both increased TB-associated IRIS and TB-associated IRIS-related mortality when compared to later ART. It did not show increased mortality with early ART.

Furthermore, in the discussion, the authors concluded that ‘In this regard, both TB-IRIS and deaths due to TB-IRIS favor late treatment of HIV among TB-HIV co-infected patients'. This conclusion is erroneous and based on another approximation in the manuscript. Specifically, the authors wrote that the number of deaths directly attributed to TB-associated IRIS was 9 with early ART and 0 with late ART. However, in the appendix of the quoted TB-HAART trial (Table S5), it is noted that 1 death in the early ART group and 6 deaths in the late ART group were attributed to TB-associated IRIS [2]. In the quoted trial of Amogne et al., TB-associated IRIS was also the cause of death in 1 case, however with no treatment timing noted [3]. Even if this latter death represented an early ART IRIS-related death, it would result in a total of 11 and 6 deaths attributed to IRIS in early and late ART groups, respectively, rather than 9 vs. 0. As well, the authors did not take into account data from these 2 trials out of the 8 they evaluated in the meta-analysis with no explanation for this omission.

Early ART remains a critical intervention in TB/HIV disease, especially in those individuals with CD4 cell counts below 50/mm^3^ because mortality in this group is particularly high and is significantly mitigated by early ART [4]. Challenging the overall survival advantage of early ART by the authors is not supported by the analysis they presented in this paper. Indeed, Chelkeba L. et al. show that early ART initiation has a significant impact on saving lives despite being associated with increased TB-associated IRIS events, few of them fatal, consistent with conclusions of previous studies and with the 2016 WHO guidelines (‘While there was a statistically significant increase in IRIS-related mortality associated with early ART, the number of deaths was small in comparison with overall deaths') [4].

## Conflicts of interest

None declared.

## Provenance and peer review

Not commissioned, externally peer reviewed.

## Ethical approval

N/A.

## Sources of funding

No funding source.

## Author contribution

FXB drafted the first version of the paper. DL and AEG improved the manuscript. All 3 authors approved the final version of the paper.

## Consent

N/A.

## Registration of research studies

N/A.

## Guarantor

All 3 authors are Guarantor for this paper.

## Declaration of competing interest

FXB, DL and AEG conducted the CAMELIA clinical trial (N Engl J Med 2011; 365:1471–81), one of the 8 trials analyzed by Chelkeba L. et al.

No other interest declared.
